# Doping free transfer of graphene using aqueous ammonia flow

**DOI:** 10.1039/c9ra06738h

**Published:** 2020-01-06

**Authors:** Morteza Hassanpour Amiri, Jonas Heidler, Ahmar Hasnain, Saleem Anwar, Hao Lu, Klaus Müllen, Kamal Asadi

**Affiliations:** Max-Planck Institute for Polymer Research Ackermannweg 10 55128 Mainz Germany asadi@mpip-mainz.mpg.de; School of Chemical & Materials Engineering, National University of Sciences & Technology Sector H-12 Islamabad Pakistan

## Abstract

Doping-free transfer of graphene produced by catalytic chemical vapor deposition (CVD) on copper foil, is still a technical challenge since unintentional doping of the transferred graphene layer yields an uncontrolled shift of Dirac point in graphene-based field-effect transistors (FETs). Typically, CVD graphene is released from the growth template by etching of the template, *i.e.* copper. During the etching process, ions adhere to the graphene layer resulting in unintentional doping. We demonstrate that washing a CVD graphene layer in an aqueous ammonia flow bath after etching copper, removes the majority of the unintentional dopants. FETs fabricated from graphene after washing in DI-water display a large scattering in Dirac bias with lowered mobility. In contrast, FETs from graphene that is washed in ammonia furnish better performance with high geometrically normalized mobility exceeding 2.4 × 10^4^ cm^2^ V^−1^ s^−1^, balanced transport and a Dirac voltage near zero. We attribute the improved FET behavior to effective removal of the ions with a typical density of 4 × 10^12^ cm^−2^ from the graphene layer.

## Abstract 2

The paper addresses the technical challenge of producing doping-free transferred graphene layers produced by catalytic chemical vapor deposition (CVD), thereby preventing uncontrolled shift of the Dirac point in comprising field-effect transistors.

## Graphical abstract

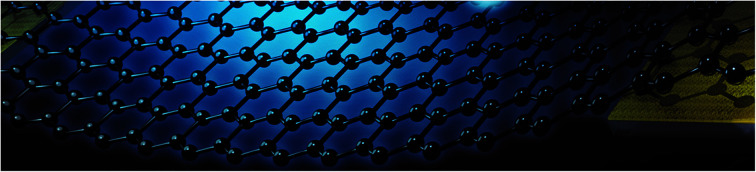

Two-dimensional (2D) materials such as graphene have been extensively studied for various electronic and opto-electronic applications.^1–5^ Several methods have been proposed for wafer-scale production of graphene monolayer, among which catalytic chemical vapor deposition (CVD) of graphene is a technologically viable method.^6,7^ To release the graphene layer, a sacrificial polymeric layer, typically poly(methylmethacrylate) (PMMA) is spincoated on top of an as grown graphene layer, followed by etching of the copper template. To fabricate electronic devices, such as graphene field-effect transistors (FETs), the graphene/PMMA stack is transferred onto a secondary substrate. FETs are commonly realized by patterning the source and drain electrodes on top of the graphene layer, or by transferring, the graphene/PMMA layer onto a substrate with pre-patterned source and drain electrodes.

Graphene transfer is one of the critical issues in the fabrication of graphene FETs and their upscaling.^8^ Since FETs are sensitive interface devices, the presence of ions (or any ionic species) at the interface between the graphene layer and the gate dielectric leads to unintentional doping of the graphene layer, which drastically affects the electrical performance.^9–11^ As a result, the Dirac point shifts to voltages much larger than 0 V so that instead of an ideal ambipolar behavior, the graphene FET exhibits unbalanced electron/hole transport or even a seemingly unipolar behavior.

Herein, we demonstrate CVD-based graphene FETs with balanced ambipolar electron/hole transport. Towards that end, we have introduced an extra washing step of graphene in aqueous ammonia after etching away the copper.^12,19^ The FETs are fabricated using the conformal transfer technique.^8^ Ammonia effectively removes the majority of ions (nearly 4 × 10^12^ cm^−2^ dopants) that otherwise would unintentionally dope the graphene layer. The extra washing step also improves the mobility of the charge carriers and shifts the position of the Dirac point to biases close to zero volt. We attribute the improved FET behavior to effective removal of Cu^2+^ from the graphene layer, and quantify the density of ions.

The process flow for graphene transistor fabrication is schematically shown in Fig. 1a. CVD graphene (Graphenea) on Cu foil (25 μm thickness, 99.8%) is coated with PMMA (Aldrich). To minimize errors due to batch-to-batch variations, all the experiments presented here are conducted using graphene obtained from small cuts of the same Cu foil. PMMA is dissolved in toluene (Acros) with 10 wt%, and filtrated through 1 μm filters prior to spincoating (all materials are used as received). The thickness of the PMMA layer varies from 700 to 800 nm. The graphene layer on the uncoated surface of the copper foil is removed using an oxygen plasma. The Cu foil is etched in 1 molar aqueous bath of FeCl_3_ and the floating graphene/PMMA layer is thoroughly washed with DI water. The “ammonia” treatment is performed by placing the floated graphene/PMMA film in a continuous flow bath of NH_3_ : DI-water (1 molar) for 30 min followed by a drying step at 40 °C for 12 hours in a vacuum oven at 10^−1^ mbar. Hereafter, we use w-graphene and a-graphene to refer to graphene that is washed in DI-water or aqueous ammonia flow bath, respectively. Subsequently, the graphene/PMMA layers are transferred to a pre-patterned Si-wafer using the conformal transfer technique. After evaporation of residual water, the PMMA layer is removed in a fresh acetone bath. Subsequently, the exposed transferred graphene is washed in a deionized water bath. The final cleaning processes are repeated several times to minimize the PMMA debris. We note optimizing the transfer process toward full removal of the PMMA layer or minimizing structural defects were beyond the scope of this work.

**Fig. 1:**
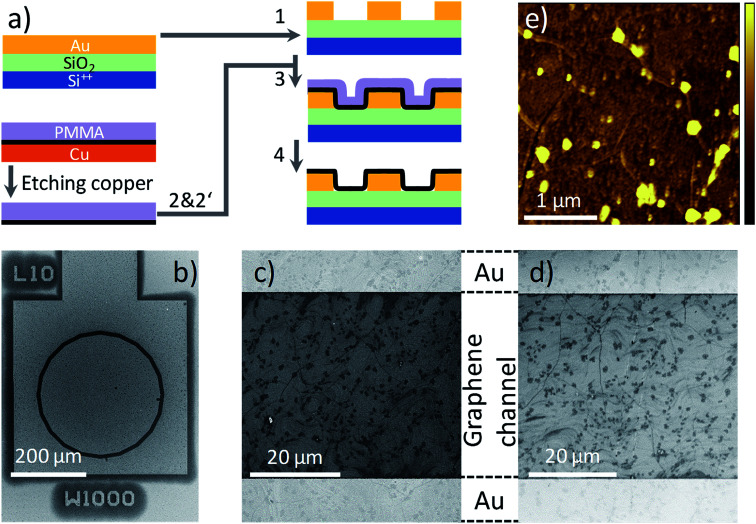
(a) Process flow for the fabrication of the graphene transistors. (1) The electrodes are patterned on the substrate. After etching copper template, graphene/PMMA is washed in DI-water, (2) or DI-water and subsequently in aqueous ammonia, (2′) and then conformal transferred onto the substrate (3). In (4), PMMA is washed away. (b) SEM images of a transistor and one of the CTLM test structures with a channel width of 1000 μm and channel length of 10 μm after coating with the graphene layer. (c and d) SEM images of w-graphene and a-graphene, respectively, transferred onto a pre-patterned FET test structure. The bright region on the top and bottom are the Au contacts of the test structure, and the area in between the contacts is uniformly covered with the graphene layer. (e) AFM topography images of the a-graphene after washing in ammonia flow bath. The topography scale bar is from 0 to 15 nm. The bright spot are due to PMMA debris.

The pre-patterned substrates are fabricated on 6-inch Si wafers with 250 nm of thermally grown SiO_2_. The electrodes are fabricated by sputter deposition of Ti/Au (2 nm/150 nm), and patterned by conventional photolithography. The spacing between the source and drain electrode varies from 1 μm to 40 μm at a fixed channel width of 1000 μm, having a concentric geometry. A scanning electron microscopy (SEM) image of the transistor test structure is given in Fig. 1b. A The SiO_2_ surface is passivated with hexamethyldisilazane (HMDS) prior to conformal transfer of graphene/PMMA. The graphene transferred onto a pre-patterned substrate is inspected using SEM. Although the pre-patterned substrates have an uneven topography, the SEM image presented in Fig. 1c–d, provides evidence that the graphene morphology on the Au contact and the SiO_2_ layer are identical and extrinsic defects such as cracks or film discontinuities are absent. Importantly, the same morphologies are observed for both w- and a-graphene. Both graphene layers showed some PMMA debris. A typical atomic force (AFM) topography image for the a-graphene layer is shown in Fig. 1e. The same AFM images are obtained also for the w-graphene (not shown here). From both TEM and SEM images it is clear that washing the graphene layer in an ammonia flow bath does not harm the integrity of the graphene layer. Presence of the PMAA layer during the ammonia washing and transfer steps is helpful for the structural integrity of the graphene.

Optical microscopy and atomic force microscopy (AFM) (Nanoscope Dimension 3100 Bruker) were used to analyze the surface morphology of the transferred graphene films before and after ammonia treatment. X-ray photoelectron spectroscopy (XPS) was conducted with a Kratos Axis Ultra DLD spectrometer (Kratos, Manchester, England) using Kα excitation of an Al source with a photon energy of 1487 eV. The data was acquired in the hybrid mode with a beam spot size of 300 × 700 μm^2^ using a 0° take-off angle, defined as the angle between the surface normal and the axis of the analyzer lens. A charge neutralizer was used during spectra collection to compensate sample charging. Survey and detailed region XP spectra were collected by setting analyzer pass energy at 80 eV, C 1s and O 1s high-resolution spectra were collected with an analyzer pass energy of 20 eV. A neutralizer was always used during spectra collection. No X-ray damage was inferred to the sample during measurements. Atomic compositions were calculated by subtracting a linear background from the collected spectra with setting an analyzer pass energy at 80 eV. The peak areas were normalized by the manufacturer supplied sensitivity factors, and atomic compositions were calculated accordingly with CasaXPS software.

The sheet resistance of the graphene layer, before and after ammonia treatment is determined using a circular transfer line test method (CTLM), as detailed in ref. 8. The sheet resistance is obtained by forcing different constant currents (ranging from 1 μA to 100 μA), and measuring the voltage drop. In total 32 test structures are measured. The extracted sheet and contact resistances are constant for different current levels.

Metal/insulator/graphene/metal (MIS-) diodes are fabricated by deposition of Cr/Au (1 nm/50 nm) on to thoroughly cleaned glass substrates. After deposition of the graphene/PMMA layer on the substrates and subsequent drying, 100 nm of a Au top contact are evaporated through a shadow mask. The PMMA layer thickness was 700 to 800 nm. The capacitors had a device area of 0.16 mm^2^. For each case, 25 MIS-diodes are fabricated and the results are averaged. All electrical measurements were performed in high vacuum (10^−6^ mbar) using a Keithley 4200 semiconductor characterization system and a Keysight 4980 precision LCR meter.

First, the sheet resistance, *R*_sh_, of the graphene layer after different washing processes are determined from the CTLM test structures.^8^ The *R*_sh_ for w-graphene amounts to 450 Ω □^−1^ and remains constant for different current levels whereas the a-graphene layer shows an increased *R*_sh_ of 500 Ω □^−1^. Observation of higher *R*_sh_ is due to the de-doping of the graphene layer.

Transfer characteristics of the FET fabricated with w-graphene is presented in Fig. 2. The w-graphene FET exhibit a large shift in Dirac voltage towards positive voltage, and an unbalanced transport leading to a “seemingly” unipolar p-type instead of an ambipolar-like behavior. Both observations indicate that the w-graphene layer is doped with positively charged ions. Presence of ions screens the gate bias and shifts the Dirac voltage to large positive voltages. Therefore, we attribute the unipolar characteristics of the w-graphene FET to unintentional doping of the graphene layer during the transfer process.^13–16^ This can be caused by copper, water or etchant (FeCl_3_ in our case) ions. A doping/de-doping mechanism will be discussed later.

**Fig. 2:**
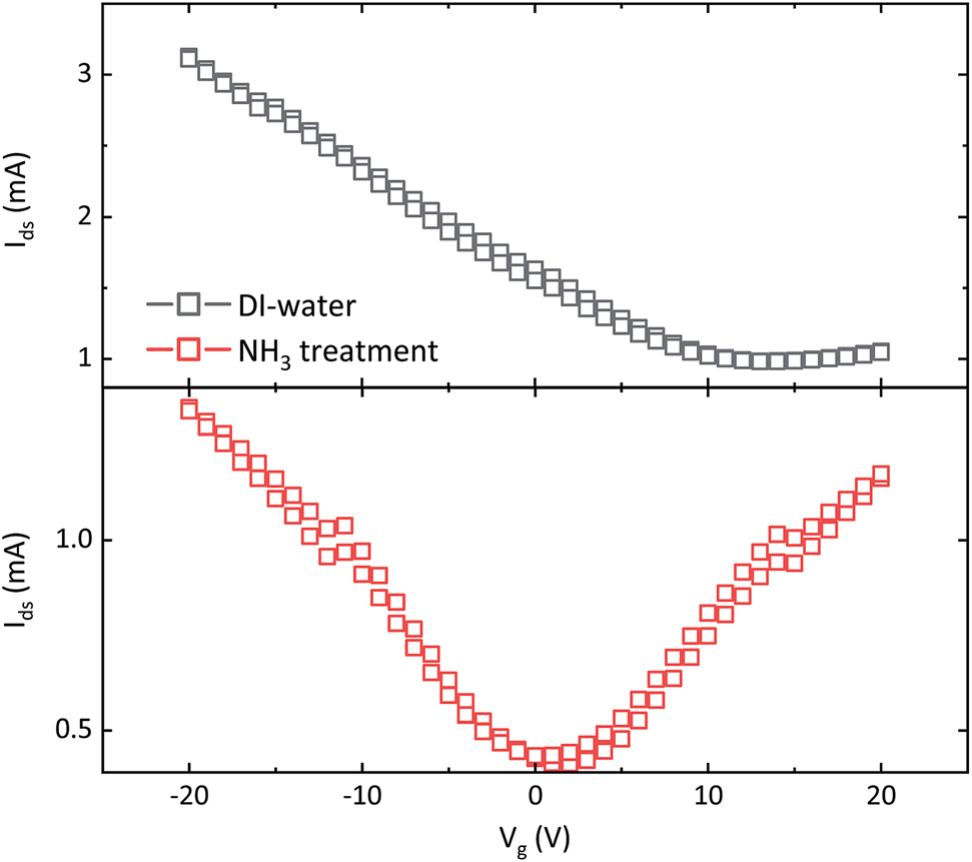
Transfer characteristics of FETs fabricated with graphene layers that are washed only in DI-water (top) and DI-water plus aqueous ammonia flow (bottom). Both transistors have channel length and width of 10 and 1000 μm, respectively. The bias on drain electrode is +100 mV. Source electrode is grounded.

To eliminate the troublesome unintentional doping, and recover ambipolarity in the graphene FETs, we have introduced an extra washing step in ammonia flow bath, right before transfer of graphene/PMMA layer onto the FET substrate. The FET transfer characteristics of the resulting a-graphene FET is presented in Fig. 2. Interestingly, the FET shows a shift of the Dirac voltage to a gate bias near zero volt, namely 1–2 V. Moreover, the transistor shows a nearly symmetric transfer curve. This indicates a balanced charge carrier concentration in the graphene layer, *i.e.* the Fermi level is near or at the Dirac point, which means that ions, or charged species are absent. It should be added both transistors presented in Fig. 2 are fabricated from the same batch of CVD graphene, coated with PMMA.

It has been recently demonstrated that using paraffin instead of PMMA for the graphene transfer yields four-fold increase in field-effect mobility, *μ*_FE_, of the graphene layer.^17^ Since *μ*_FE_ is a device parameter that is affected by the geometrical parameter *W*/*L* (channel width/length). For a fair comparison, here use a normalized mobility, *μ*_n_, which is the *μ*_FE_ multiplied by the geometrical parameter *W*/*L*:
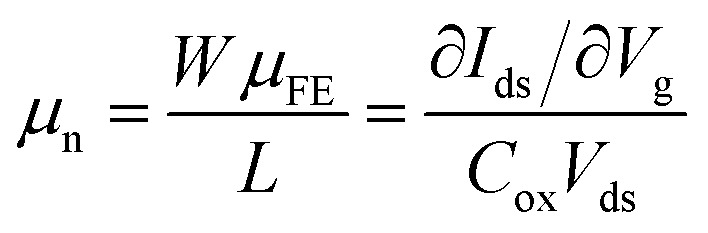
where *C*_ox_ is the capacitance of the gate dielectric. The geometrical normalization allows for a fair comparison between the mobilities obtained from the state-of-the-art paraffin-enabled graphene FETs and those presented in Fig. 2. The maximum calculated *μ*_n_ for the paraffin transferred graphene amounts to 6.5 × 10^3^ whereas *μ*_n_ for a-graphene amounts to 2.6 × 10^4^, which is by a factor of 4 larger.

The current in the a-graphene FET is lower than the one in the w-graphene FET. This indicates that the ammonia treatment de-dopes the graphene layer, and reduces the number of residual carrier in the graphene layer. We suggest a tentative de-doping mechanism as follows. Aqueous FeCl_3_ is a well-known etchant for copper, and a strong Lewis acid. The chloride plays an essential role in dissolving copper since Cl^−^ is a strong complexing agent for Cu^2+^ ions. The etching reaction is written in a simplified form as:

1 2Fe^3+^ + Cu → 2Fe^2+^ + Cu^2+^

The Cu ions in the form of CuCl_2_ are soluble in water. As a result there can always be traces of Cl^−^ or Cu^2+^ ions in w-graphene. The extra washing step of graphene in an ammonia flow bath is ideal to remove both ions. The NH_3_ molecules extract the residual Cl^−^ ions from the graphene layer as ammonium chloride, which is soluble in water and is washed away. Furthermore, the Cu^2+^ ions form an ionic complex in water, namely hexaaquacopper(ii) ion [Cu(H_2_O)_6_]^2+^. The ammonia acts simultaneously as ligand for the [Cu(H_2_O)_6_]^2+^ ions to form solid precipitates of the neutral complex according to the following reaction:

2 [Cu(H_2_O)_6_]^2+^ + 2NH_3_ → [Cu(H_2_O)_4_(OH)_2_] + 2NH_4_^2+^

Therefore, the extra ammonia washing step would also remove Cu^2+^ ions that can potentially act as dopant.

To unambiguously demonstrate the presence of ions and further estimation of their surface concentration we have performed XPS measurement for the graphene layer before and after ammonia transfer. A closer up X-ray photoelectron spectra^18^ for C 1s, Cu 2p, Fe 2p and Cl 2p for both w- and a-graphene are presented in Fig. 3. The C 1s spectrum of the graphene does not change before and after ammonia treatment, indicating that ammonia does not attack the C=C bonds. The Fe 2p spectra shows that washing the graphene layer with DI-water is sufficient to effectively remove Fe ions, as the XPS spectra does not show presence of Fe before and after ammonia flow bath treatment. The Cu 2p spectra clearly shows a weak signal near 930 eV, which could be assigned to Cu (2p_3/2_) electrons. After the ammonia washing step, the weak signal has disappeared in the background noise. Therefore, washing of the graphene in the ammonia flow bath has removed minute traces of Cu ions. The difference in XPS spectra is more pronounced for the Cl 2p survey of w- and a-graphene. While the a-graphene does not show presence of Cl, the w-graphene clearly shows a peak at ∼199–200 eV. The amount of Cl for the w-graphene and a-graphene is calculated, which amount to 0.27 ± 0.04% and 0.16 ± 0.04% respectively. Therefore using an ammonia flow bath treatment has effectively removed majority of the Cl ions.

**Fig. 3:**
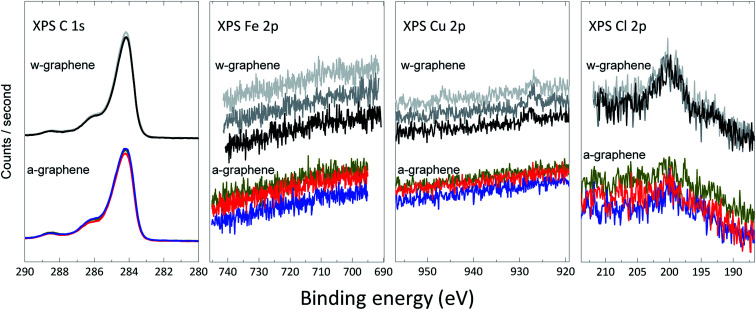
High-resolution C 1s, Fe 2p, Cu 2p, and Cl 2p XPS spectra for w-graphene (top) and a-graphene (bottom). For every graphene layers, three separate samples are measured.

To estimate the concentration of ions, we have fabricated MIS-diodes. A constant DC bias of +10 V superimposed with an AC signal of 0.1 V with variable frequency is applied to the Au electrode contacting PMMA. The capacitance–frequency responses of the MIS-diodes are given in Fig. 4. MIS-diodes from w-graphene show a decreasing capacitance with increasing frequency. The capacitance at 100 Hz is 160 pF which reduces to 130 pF at 100 kHz. The reduction in capacitance is directly related to ion mobility, because at low frequencies, the ions can follow the changes in the electric field, while at high frequency they are static. In sharp contrast, the MIS-diodes fabricated with a-graphene possess a lower capacitance of ∼30 pF that does not vary with the excitation frequency. A constant capacitance over a large frequency range is a clear indication that ions are absent in the MIS-diode stack. Since both MIS-diodes have similar PMMA thickness, we can calculate the difference in the number of excess positive ions/charges in w-graphene by considering the capacitances measured at 100 kHz. Taking 130 and 30 pF for w- and a-graphene respectively, we have estimated an areal doping density of 3.9 × 10^12^ cm^−2^ for w-graphene. The doping density is nearly the same as the intrinsic carrier density in graphene. As a result, a nearly two fold increase in the current of the transistors is expected when we assume that the mobility remains constant. Interestingly, the currents at *V*_g_ = −20 V for the transistors presented in Fig. 2 differs by a factor of 2.

**Fig. 4:**
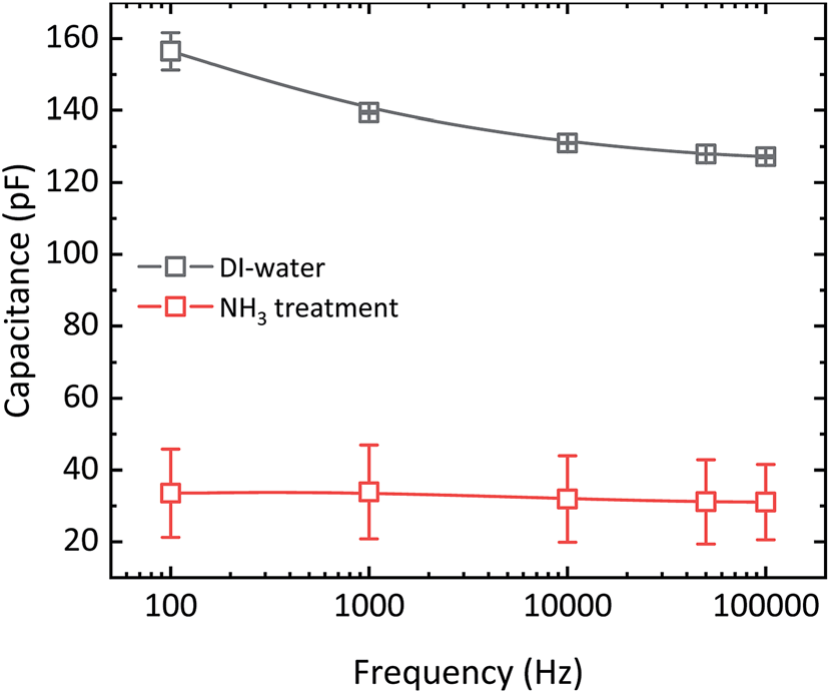
Frequency response of the MIS-diodes fabricated with graphene layers that are transferred after washing in DI-water and aqueous ammonia. Every data point is an average of 5 different devices. The bias applied to the Au electrode contacting PMMA is 10 V. The amplitude of the AC signal is 0.1 V. The MIS-diodes have an area of 0.4 × 0.4 mm^2^. The solid line is drawn as a guide to the eye.

In summary, washing of the graphene layer after etching copper in ammonia flow bath is a crucial step to achieve FETs with balanced transfer characteristics and with zero Dirac voltage. The ammonia can effectively remove both chlorine and copper ions. We note that the PMMA transfer technique is not ideal for the realization of high quality graphene FETs. Therefore, upon combination of ammonia flow bath with more advanced transfer techniques, high performance CVD-graphene based FET are within reach.

## Conflicts of interest

There are no conflicts to declare.

## Supplementary Material
